# The role of parents in the motivation of young athletes: a systematic review

**DOI:** 10.3389/fpsyg.2023.1291711

**Published:** 2024-01-08

**Authors:** Zhendong Gao, Chen Soon Chee, Mohd Rozilee Wazir Norjali Wazir, Jiaxu Wang, Xiaojian Zheng, Tao Wang

**Affiliations:** ^1^Department of Sports Studies, Faculty of Educational Studies, Universiti Putra Malaysia, Serdang, Selangor, Malaysia; ^2^Department of Foundations of Education, Faculty of Educational Studies, Universiti Putra Malaysia, Serdang, Selangor, Malaysia

**Keywords:** young athletes, motivation, sports parenting, parenting styles, parenting practices

## Abstract

**Objectives:**

Parents are one of the main social agents that shape young athletes’ experiences and participation in sports, but they are also the least explored group in the literature. Therefore, the purpose of this study was to conduct a systematic review of research on the role of parents in the motivation of young athletes.

**Method:**

The systematic literature review consisted of four electronic databases from which 29 articles published in English and in full-text form in peer-reviewed journals between 1999 and 2023 were retrieved.

**Results:**

A total of 29 studies met the eligibility criteria. These studies collectively surveyed 9,185 young athlete participants and 2,191 parent participants. The sample comprised 26 quantitative studies and 3 qualitative studies. The findings underscore that parents play both unique and synergistic multidimensional roles in motivating young athletes. Parents’ positive goals and values, autonomy-supportive parenting styles, moderate parental involvement, positive parent–child relationships, and a parent-initiated task climate are identified as optimal parenting strategies.

**Conclusion:**

While parents undeniably play a crucial role in motivating young athletes, the manner and extent of their involvement are key.

## Introduction

Motivation is generally considered to involve the onset, direction, intensity, and persistence of individual behavior (Robbins et al., 2013). As a complex construct, it is not an observable entity, making it challenging to accurately conceptualize and measure (Lavallee et al., 2012). For a long time, motivation has been a critically important factor in the field of sports (Rodrigues et al., 2020), and has continuously attracted widespread attention from researchers in contemporary sports and sports psychology (Rodrigues et al., 2020; Borg and Willoughby, 2022; Castillo-Jiménez et al., 2022). The motivation of young athletes refers to the internal or external factors that drive them to participate, continue participating, or show enthusiasm and effort in a particular sporting activity (Roberts and Treasure, 2012). It encompasses psychological, social, and environmental factors that contribute to the initiation, maintenance, and intensity of athletes’ participation. In summary, motivation plays a crucial role in shaping the attitudes, behaviors, and perseverance of young athletes in their participation in sports (Teixeira et al., 2012; Martins et al., 2017; Mallia et al., 2019).

Within the framework of social ecological theory, adolescent development is a complex system influenced by multiple layers of the surrounding environment (Bronfenbrenner and Morris, 2007). Although parents, coaches, and peers all contribute to the outcomes of young athletes in this context, parents remain the primary social agents shaping adolescent experiences and participation in sports throughout childhood and adolescence (Harwood and Knight, 2015). As early as 2004, Fredricks and Eccles (2004, p. 145) posited that “parents play a significant role in children’s early sports socialization.” Parents provide essential material, emotional, organizational, and financial support to ensure their children can engage in sports activities (Wolfenden and Holt, 2005; Holt and Knight, 2014). Critical literature reviews also reveal that the type of support provided by parents, their parenting styles, self-emotional needs, relationships with others, involvement in organizational management, and meeting the dynamic needs of young athletes at different stages of their sports journey are all aspects that a successful sports parent should address (Harwood and Knight, 2015). Thus, it is evident that parents have a significant influence on youth sports in various aspects.

In research exploring the impact of parents on youth sports, there has been an increase in the understanding and application of motivation theories. These include Harter’s (1978) Competence Motivation Theory (CMT); Achievement Goal Theory (AGT) by Nicholls (1984); Self-Determination Theory (SDT) by Deci and Ryan (1985); and Expectancy-Value Theory by Eccles et al. (1983).

Competence Motivation Theory (CMT) suggests that an individual’s perception of their ability in any achievement domain is a key component of their motivation to achieve in that domain (Harter, 1978), meaning that the internal drive of an individual is the pursuit of a sense of ability. Moreover, Harter (1978) noted that feedback and behavior from significant others could play a vital role in the socialization and cognitive development of children and adolescents. For example, if parents have a strong belief in their own sporting abilities, their children are likely to have a higher evaluation of their own sporting abilities (McCullagh et al., 1993). Studies show that parents’ role modeling in sports, positive feedback on children’s sports performance, and belief in their children’s sports abilities can significantly influence children’s perceptions of their abilities (Babkes and Weiss, 1999).

Achievement Goal Theory (AGT) posits that individuals develop either a fixed or incremental cognition of their abilities in achievement domains. These differing perceptions, in turn, affect their motivation (Nicholls, 1989), manifesting as either a task-oriented or ego-oriented goal. When motivation is derived from a task-involved climate, it leads to higher levels of effort and enjoyment in practice (Bardach et al., 2020), whereas when it is ego-oriented, it indicates participation for greater social recognition and value (González Valero et al., 2017). In recent years, Achievement Goal Theory has evolved from its initial simple dichotomy model (mastery goals vs. performance goals) to a more complex multidimensional model, such as the 3×2 model, which further subdivides goals into six types based on the focus of the goal (task, self, others) and approach (approach, avoidance) (Elliot and McGregor, 2001; Elliot et al., 2011). Research has found that parents (and other significant individuals) can create or initiate a motivational climate that influences children (Chan et al., 2012).

Self-Determination Theory (SDT) emphasizes the types of motivation behind individual behaviors and how these motivations affect a person’s actions and psychological health (Ryan and Deci, 2000; Deci and Ryan, 2008). It is a motivational process theory about human self-determined behavior, explaining the extent to which people reflect an identification with their actions and an understanding of their choices. It underscores the role of social and environmental factors in influencing individual motivation (Ryan and Deci, 2000; Deci and Ryan, 2008). SDT helps establish a theoretical understanding of the influence of parents on children’s autonomous motivation (Ryan and Deci, 2000). This framework identifies three basic psychological needs: autonomy, competence, and relatedness. Research shows that athletes experience satisfaction when they feel the source of their actions, effectiveness in achievement, or proficiency and satisfying interpersonal relationships. Conversely, they experience frustration when they feel pressured, alienated, or face unattainable tasks (Ryan and Deci, 2000).

Expectancy-Value Theory suggests that individuals choose and persist in tasks based on their expectations of success (confidence in accomplishing a task) and the value of the task (the task’s attractiveness and importance to the individual) (Eccles et al., 1983; Eccles and Wigfield, 2002). The theory particularly emphasizes the intrinsic value of tasks (interest and enjoyment), utility value (practicality for future goals), achievement value (personal satisfaction from completing the task), and costs (effort and sacrifices required to complete the task) (Leaper, 2011). In the context of youth sports, Expectancy-Value Theory is used to understand how parents’ attitudes and beliefs relate to their children’s attitudes, beliefs, and values in sports (Fredricks and Eccles, 2004; Horn and Horn, 2007). The theory mentions that children’s behavioral choices are based on their expectations of success in a task and its perceived importance and value, and the perception of significant others’ (like parents) beliefs can influence their expectations and values (Eccles et al., 1983). Research indicates that there is a link between children’s perceptions of the value their parents place on sports and the children’s perceived competence and value attributed to the activity (Eccles and Harold, 1991; Fredricks and Eccles, 2002).

In previous research, we observed that most studies on adolescent sports focusing on Achievement Goal Theory (AGT) (Harwood et al., 2015) and Self-Determination Theory (SDT) (Chu and Zhang, 2019) primarily concentrate on the motivational climate created by coaches, with scant attention given to the atmosphere fostered by parents. Although coaches are undeniably key social agents in sports, it seems that in some aspects, parents’ influence on the motivation of young athletes has surpassed that of coaches (O’Rourke et al., 2014; Atkins et al., 2015; Amorose et al., 2016). These findings highlight the critical and unique role of parents in youth sports. Currently, there is a lack of focused attention and analysis on the impact generated by parents as social agents. Therefore, we believe that a targeted systematic review of the literature on the influence of parents as a subject is necessary. With this in mind, the purpose of this study is to systematically review and analyze the correlation between parental influence variables (such as the motivational climate created by parents, goals and values, parenting styles, and parenting behaviors) and young athletes’ motivational variables (achievement goals, self-determination motivation, competence, values) based on the four motivational theory frameworks. The aim is to identify the best parental intervention strategies in sports and to provide directions for future research and practice for coaches, sports psychologists, organizations, and researchers.

## Methods

This review is reported under the Preferred Reporting Items for Systematic Evaluation and Meta-Analyses (PRISMA) (Moher et al., 2015).

### Search strategy

The literature search was conducted across four internationally recognized databases: Web of Science, Scopus, SPORTDiscus, and PsycINFO. These databases have gained academic credibility and have been utilized in previous systematic reviews concerning sports and sports psychology (Norris et al., 2017; Tessitore et al., 2021). The systematic literature search was carried out in February 2023. Given that prior research has indicated that earlier studies largely lacked theoretical grounding and a nuanced understanding of outcomes, the search was restricted to peer-reviewed journal articles published in English between January 1999 and February 2023. Excluded from the search were abstracts, conference proceedings, dissertations, book chapters, and articles published in non-peer-reviewed journals. The search level for each database encompassed title, abstract, and keywords.

In consultation with co-authors, the following combination of keywords and Boolean operators was formulated: “sport*” OR “youth-sport*” OR “youth athlete*” AND “motivat*” OR “psychological-need*” AND “famil*” OR “parent*” OR “father*” OR “mother*.”

### Inclusion/exclusion criteria

The research question of this study is the association between parents and the motivation of young athletes. Therefore, the *a priori* eligibility criteria include: (a) Focus on the relationship between parents and youth sports (Yes/No); (b) Inclusion of current or former young athletes with competitive sports experience (Yes/No); (c) Exclusion of participants from special populations (e.g., physical or mental illness); (d) Focus on the motivation of young athletes; (e) The study must use quantitative, qualitative, or mixed-methods designs (not reviews) and be able to provide information on the influence of parents on at least one type of motivation of young athletes (quantitative: e.g., correlations; qualitative: e.g., categories). The subjects of this review are young athletes, but the included studies must primarily involve athletes in the adolescent stage (10–19 years old) to emphasize the association between parents and the motivation of young athletes during adolescence.

### Sifting of retrieved citations

**Figure 1:**
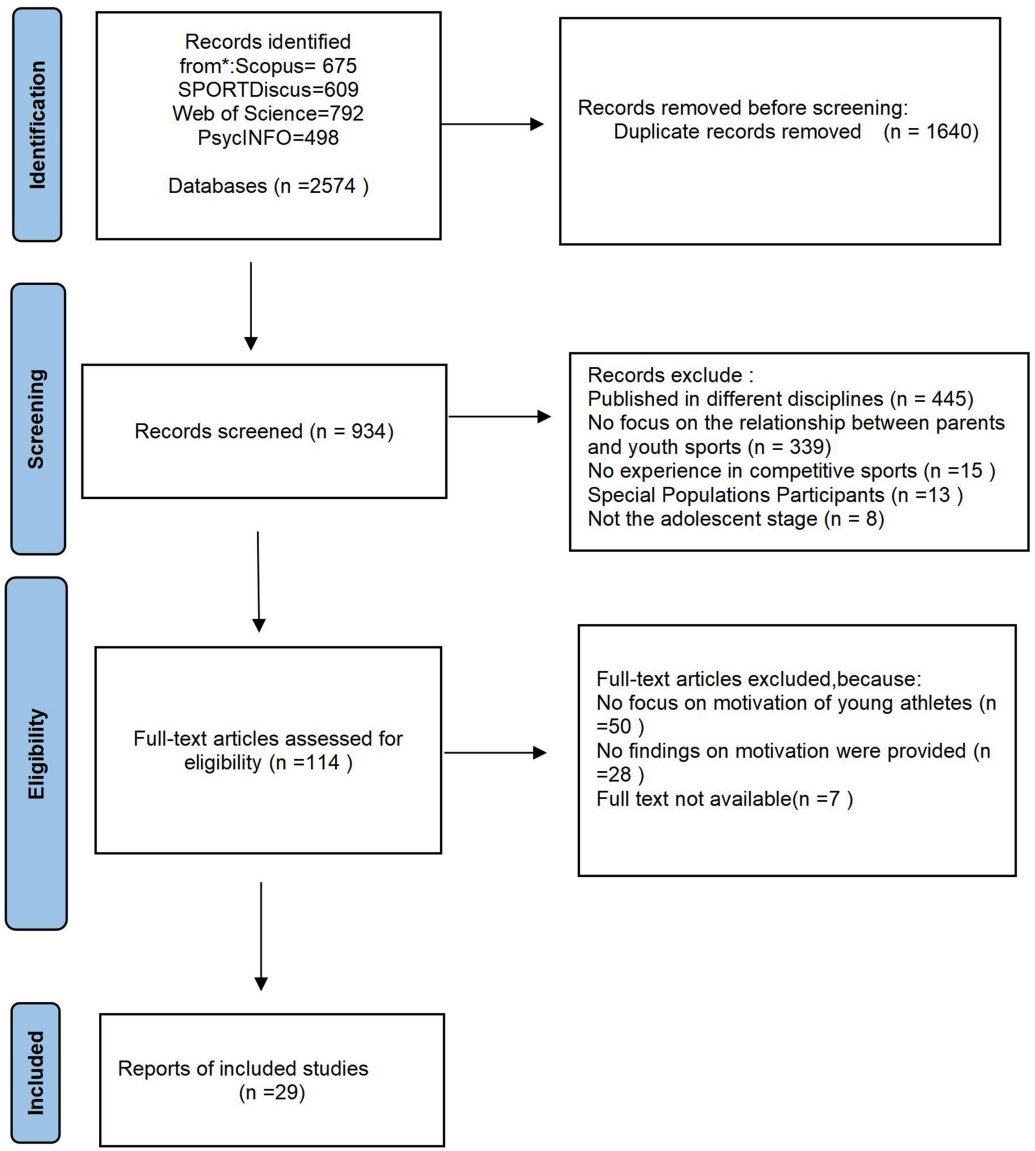
PRISMA flowchart showing how the final sample of 29 studies was obtained (applying inclusion/exclusion criteria).

This study adheres to the guidelines of the Preferred Reporting Items for Systematic Reviews and Meta-Analyses (PRISMA) (Moher et al., 2015). The search process, as illustrated in Figure 1, involves a hierarchical assessment. Initially, the literature retrieved from the search was downloaded to Zotero for study selection based on titles and abstracts, after removing duplicates. Subsequently, the full texts of the remaining studies were retrieved and assessed for eligibility. If necessary, both abstracts and full texts were screened. Any discrepancies regarding the inclusion of specific studies were resolved through consensus meetings. In cases where consensus was not reached, a third researcher made the final decision on inclusion or exclusion. The first author conducted each search, and basic information (i.e., authors, publication year, and article title) of each retrieved article was recorded in a Microsoft Excel® spreadsheet to ensure comprehensive audit tracking. The initial search yielded 2,574 published papers, and after removing duplicates, 934 papers remained. A total of 445 papers were further excluded for being published in different disciplines. Another 339 papers were excluded for not focusing on the relationship between parents and youth sports. Thirty-six papers were excluded due to lack of competitive sports experience, participation of special population groups, and primary subjects not being adolescents. Of the remaining 114 papers, 85 were further excluded because they did not focus on the motivation of young athletes, did not provide results about motivation, or did not have the full text available. As a result, 29 papers were included in this systematic review.

### Data extraction and quality assessment

Upon completion of the data search, data were extracted from eligible studies using a predetermined form that included: (1) authors and publication year; (2) study design and methodology (e.g., longitudinal, cross-sectional, quantitative, qualitative); (3) participant characteristics (gender, age, and type of sport for both parents and young athletes); (4) modes of parental influence (Parent-initiated motivational climate, goals and values, participation behavior, parenting style); (5) theoretical framework; (6) key findings. We employed a narrative synthesis approach to review and amalgamate the results of each study, a technique conducive to presenting the relevant information, connections, and structure of the research findings effectively (Popay et al., 2006). The first author abstracted the data into a standardized form, which was then checked by the fourth author (see Table 1).

**Table 1:** Study characteristics of studies included in final sample.

Authors	Aim(s)	Design	Participants	Country	Sport level	Ways of parental influence	Theoretical framework(s)	Key findings
Babkes and Weiss (1999)	Investigated the relationship between children’s perceptions of parental influence and their psychosocial responses to participation in competitive football	Quant/Cross-Sectional	227 youth athletes (114F/113M,Mage = 10.6,SD = 54AR = 9–11.6), parents (160F/123M)	United States	Competitive all-state football program	Parental attitudes and involvement behaviors	CMT	Parents who were seen as positive sporting role models, who had more positive beliefs about their child’s abilities and who gave more frequent positive incidental responses to performance success were associated with athletes with higher perceived competence, enjoyment and intrinsic motivation.
Given (2001)	The relationship between young people’s goal orientations and those of their coaches and parents	Quant/Cross-Sectional	90swimmers, (35 M/55 F, AR = 12–15);71Parents (24 M, 47 F)	United Kingdom	State Swimming Teams	Goal orientation between parents	AGT	Teenagers’ self-reported goal orientations were highly correlated with their perceptions of significant adult goal orientations, but not with significant adult self-reports. Teenagers believe their thoughts and feelings about swimming are influenced more by their coaches than by their parents
Gagne (2003)	(a) parental and coaching support and athletes’ enduring and daily motivation and needs satisfaction and (b) how daily motivation and psychological needs satisfaction affect athletes’ well-being in practice	Quant/Longitudinal	33 F gymnast (Mage = 13, SD = 2.35, AR = 7–18)	United States	United States Association of Gymnastics grading system 5 to 9, with a median ability level of 6	Parental autonomy support and involvement behaviors	SDT	Parental and coaching autonomy support and involvement influenced the quality of motivation of gymnasts.
White et al. (2004)	Perceived parental beliefs about the causes of success in sport: relationship to athletes’ achievement goals and personal beliefs	Quant/Cross-Sectional	183 athletes (90F/60M/30 N/aAR = 11–18) Females (Mage = 15.44, SD = 1.91); Males (Mage = 14.03, SD = 1.82)	United States	Athletes competing in team competitions at district level	Parents’ belief in success	AGT	Perceived parental beliefs were related to goal orientation and personal beliefs in a conceptually coherent manner. Parental beliefs about striving to lead to athletic success were related to the athlete’s task orientation and personal beliefs about striving to lead to athletic success.
Waldron and Krane (2005)	Investigated the combined effects of coach- and parent-initiated motivational climate on athletes’ goal orientations and changes in goal orientations during the competitive season	Quant/Longitudinal	62Youth female softball players (Mage = 14.97, SD = 0.63) Grades 9–11	United States	Junior Varsity Youth Team Members	Parent-initiated motivational climate	AGT	Athletes’ early task orientation, perceptions of task-coach-initiated climate, and parental climate with an emphasis on learning positively predicted athletes’ task orientation at the end of the season.
Ullrich-French and Smith (2006)	How adolescents’ perceptions of their relationships with parents and peers independently and in combination predict motivational outcomes in youth sport	Quant/Cross-Sectional	186 Youth football players (99 M/87F, Mage = 11.6, SD = 1.0AR = 10–14)	United States	Organized same-sex competitive travel football team (i.e., tryouts, competing in tournaments with other towns)	The relationship between parents and children	SDT&CMT&EVT	higher self-determined motivation was predicted by higher peer acceptance, quality of paternity, quality of friendship, or quality of mother–child relationships.
Papaioannou et al. (2008)	Examined the contribution of the motivational climate created by mothers, coaches and best friends in explaining differences in athletes’ achievement goals, athletic satisfaction and academic performance	Quant/Cross-Sectional	863 active athletes (488 M/372F/3N/a);420 (Mage = 14.5, SD = 0.60);430 (Mage = 11.5, SD = 0.60)	Greece	Regional sports clubs	Target orientation highlighted by the mother	AGT	All socialization agents contributed uniquely to the explained variance in athletes’ achievement goals in sport.
Keegan et al. (2010)	Qualitatively examined the motivation-related behavior of key social agents among participants in the professionalization movement	Qual/Cross-Sectional	79 sport participants (36F/43M) (Mage = 12.93, SD = 1.82, AR = 9.0–18.16).	United Kingdom	General school and football school athletes	Parental support and facilitation	SDT&AGT	The influence of coaches and parents was related to their specific roles: coaching/evaluation of coaches, support and facilitation of parents. Peers influence motivation through competitive behavior, collaborative behavior, evaluative communication and their social relationships.
Boiché et al. (2011)	The relationship between certain antecedents of parental behaviors, athletes’ perceptions of their parents’ behavior and sporting outcomes	Quant/Cross-Sectional	161 athletes (Gymnastics, Tennis & Judo) (F84/M77) (Mage = 13.8, SD = 1.77, AR = 12–16); 134 mothers (Mage = 42.8; SD = 5.93);114 fathers (Mage = 44.3; SD = 4.30)	France	Athletes in clubs competing at local, regional or national level	Parental involvement behaviors	EVT	The mother’s directive behavior can negatively affect perceived competence. The level of praise and understanding from the mother was the only significant predictor of intrinsic motivation. Perceived parental behavior significantly predicts perceived athletic ability and value of athletes
Sánchez-Miguel et al. (2013)	Examining the relationship between motivational orientation and parental behavior, including athletes’ motivational orientation, motivational climate, enjoyment and unmotivation.	Quant/Cross-Sectional	723 parents (351F/372M, M = 46.46, SD = 2.56, AR = 36–49); 723 athletes, (561 M/162F, M = 12.37, SD = 1.48, AR = 11–16)	Spain	Federation Club	Parental involvement behaviors, parental motivational orientation	SDT&AGT	Parental support for sport was positively associated with sport enjoyment and negatively associated with sport amotivation. Those athletes who felt more pressure from their parents were positively associated with sport amotivation and negatively associated with sport enjoyment. Appropriate parental involvement can contribute to athletes’ enjoyment of sport and increased motivation.
Hein and Jõesaar (2015)	Predicting young athletes’ autonomy motivation from the dimensions of perceived autonomy support from parents and coaches and peer motivational climate.	Quant/Cross-Sectional	662 young athletes (441 M/221F, Mage = 13.18 SD = 1.52, AR = 11–16).	Estonia	Estonian sports club. Participates in national and provincial competitions, but is not a member of a professional sports team.	Parental autonomy support	SDT&AGT	Autonomous support from parents and coaches as antecedents to the formation of a perceived peer motivational climate was associated with the prediction of self-determined motivation. Autonomy support from parents was a stronger predictor of self-determined motivation than autonomy support from coaches.
O’Rourke et al. (2014)	The strength of the relationship between athletes’ perceptions of the climate. Initiated by coaches and parents at the end of the season and their self-esteem, performance anxiety and intrinsic-extrinsic motivation.	Quant/Cross-Sectional	238 athletes (97 M/141F; Mage = 11.90, SD = 1.33, AR = 9–14)	United States	Regional Swimming Clubs	Parent-initiated motivational climate.	SDT	Parent-initiated motivational climate was a significant predictor of motivation related to self-esteem, trait anxiety and autonomic regulation later in the season, above coach-initiated motivational climate.
Amado et al. (2015)	How parental support/pressure affects children’s motor motivation processes and how the model operationalizes differences in gender.	Quant/Cross-Sectional	321 children (175 M/146F, Mage = 13.4, SD = 2.92, AR = 10–16);321 parents	Spain	JUDEX (Extremenian Sports Games) Clubs	parental pressure & support for basic psychological needs	SDT	Parental stress has a negative impact on the satisfaction of basic psychological needs. It also emerged as a strong positive predictor of intrinsic motivation and a negative predictor of amotivation. The results showed an average difference by gender: male athletes felt more parental pressure.
Amorose et al. (2016)	To examine the independent and interactive effects of athletes’ perceptions of autonomy support from coaches, fathers and mothers on athletes’ autonomy motivation.	Quant/Cross-Sectional	335 youth athletes (M126/F209) (Mage = 15.75, SD = 1.21, AR = 14–18).	United States	School sports team athletes	Autonomous support from fathers and mothers	SDT	Coaches’, fathers’ and mothers’ perceptions of autonomy support were all associated with athletes’ autonomy motivation. Relatively high levels of autonomy motivation were associated with the perception that at least two of the three significant others provided high levels of autonomy support.
Schwebel et al. (2016)	The effects of a motivational climate created by coaches and success criteria communicated by parents on outcome measures of anxiety, self-esteem and achievement goal orientation in post-season athletes	Quant/Cross-Sectional	612 basketball players (369 M/243FMage = 11.76, SD = 1.56, AR = 9–16)	United States	Recreational basketball league basketball players	Perceived Parental Success Standards in Sport	AGT	Parentally communicated success criteria were significantly associated with anxiety, self-esteem and achievement goal orientation in post-season athletes. Parental success criteria moderated the relationship between coach-initiated motivational climate and achievement motivation
Gaudreau et al. (2016)	How parental autonomy support and coach autonomy support relate to sport-related outcomes in young athletes.	Quant/Longitudinal	Youth footballers (*N* = 46) (F25/M21) (Mage = 12.40; SD = 0.62, AR = 11–13); gymnasts (*N* = 85F) (Mage = 12.71; SD = 2.36, AR = 9–18)	Canada	Regional U12 football team players; gymnasts competing in provincial competitions, the	Parental autonomy support	SDT	Parental autonomy support moderated the effects of coach autonomy support. Coaching autonomy support was more important at lower levels of parental autonomy support. At higher levels of parental autonomy support, coaching was less influential. Coach and parental autonomy support interacted to predict sport-related outcomes.
Weltevreden et al. (2018)	Examining the role of parents in the achievement goals of young athletes	Quant/Cross-Sectional	Football and hockey players (*N* = 140); (72 M,68F; Mage = 15.50, SD = 2.05, AR = 12–22);	Netherlands	Football and hockey club players	General parental behavior and parent-initiated motivational climate	AGT	Autonomous support and responsiveness are related to mastery goals through parental climate. Psychological control is related to performance goals through parental climate. Behavioral control is associated with mastery goals rather than performance goals. Parents appear to be more important than coaches for adolescents’ achievement goals.
Lienhart et al. (2019)	(a) examine the relationship between high-level athletes’ perceptions of parental behavior, their satisfaction of basic psychological needs and motivation to play sport, and (b) explore whether an athlete’s gender moderates these behavioral relationships	Quant/Cross-Sectional	333 French athletes (148 F/185 M, M age = 16.49, SD = 1.53 AR = 13–18)	France	Elite athletes from French intensive training centers	Parental involvement behaviors	SDT	Fathers’ perceived pressure positively predicted maladaptive outcomes (controlled forms of motivation and motivation). Mothers’ and fathers’ perceptions: praise and understanding positively predicted maladaptive athlete outcomes (self-determined forms of motivation and satisfaction with competence and relatedness), whereas mothers’ and fathers’ perceived stress negatively predicted such adaptive outcomes.
Teques et al. (2019)	Examining perceived associations with parenting practices (encouragement, reinforcement, mentoring and role models) and psychological variables (self-efficacy, social self-efficacy, self-regulation and intrinsic motivation) in athletes Elite and sub-elite young athletes.	Quant/Cross-Sectional	881 young athletes (M689, F192)Elite (*n* = 210) Sub-elite (*n* = 635) (Mage = 16.58, SD = 1.33,AR = 14–18)	Portugal	Elite athletes are classified as athletes participating in national teams. Sub-elite athletes compete at regional level	Parental involvement behaviors	SDT	Young elite athletes’ perceptions of sport-related parenting styles were correlated with their levels of mental skills and sport performance. Perceptions of parental encouragement had significantly different strong effects on intrinsic motivation compared to their sub-elite peers. Perceptions of parental role models revealed differential effects on levels of performance, intrinsic motivation and self-regulation.
Gomes et al. (2019)	(1) young athletes’ motivation to play sport is related to their parents’ behavior; (2) this relationship is moderated by cognitive assessments, even after controlling for level of competition and sporting record.	Quant/Cross-Sectional	673 young athletes. 588 M (87.4%) 85 F (12.6%), (Mage = 14.78; SD = 1.86 AR = 12–19).	Portugal	National Level 2 (436 cases, 64.8%) and Level 1 (229 cases, 34%); eight athletes did not specify their level of competition.	Parental involvement behaviors	AGT	Mothers’ behavior accounted for 15 to 16% of the variance in motivation, while fathers’ behavior accounted for 12 to 21% of the variance. The pattern of correlation varied depending on whether the athlete was assessing the behavior of the mother or the father. as cognitive assessments partially moderated the relationship between perceived parental behavior and motivation
Felber Charbonneau and Camiré (2019)	To examine parents’ and children’s perceptions of how parental involvement in sport affects the satisfaction of basic psychological needs.	Qual/Cross-Sectional	Eight parents (6 M/2F, Mage = 44;SD = 5.96, aged 36 to 53); Eight athletes (3 M/5F, M age = 14.0;SD =1.32, AR = 12–16)	Canada	Regional youth sports programs; sports level: leisure and development	Parental involvement behaviors	SDT	Parental behavior in the sporting environment was generally considered to meet the basic psychological needs of athletes, although demand frustration was also reported.
Lienhart et al. (2020)	(a) identify parents’ use of person-centered approaches and differentiated behaviors mothers’ and fathers’ behaviors; (b) explore changes in parents’ behavioral profiles throughout the season; and (c) examine future behavioral profiles affecting parents’ early season athlete scores motivation, satisfaction and frustration psychological needs at the end of the season.	Quan/longitudinal two-wave measurement	226 semi and competitive elite athletes (*F* = 90, Mage = 15.92, SD age = 1.43, AR = 12–19)	France	Elite athletes from the French Intensive Training Center, competing at regional (*n* = 73), national (*n* = 142) or international (*n* = 11) level.	Parental involvement behaviors	SDT	Three parental behavior profiles were revealed. Athletes who reported moderate levels of parental involvement at the beginning of the season scored lower in controlled motivation and autonomous frustration at the end of the season, while scoring higher in satisfaction with competence and relatedness at the end of the season than athletes in the other two profiles.
Souza et al. (2020)	Correlations between motivation, parental style and mental health in school basketball players.	Quant/Cross-Sectional	9 female basketball players Mage = 13.6SD = 0.8	Brazil	Participating in the State Games competition, school basketball players	Parenting involvement behaviors	SDT	The results demonstrate a correlation between motivation, parenting style and mental health.
De Muynck et al. (2021)	The relationship between demand-supportive and demand-hindering coaching and parenting practices and athletes’ motivation and engagement was investigated	Quant/Cross-Sectional	255 male competitive youth football players (Mage = 13.72, SD = 1.97, AR = 10–20).	Belgium	Participation in regional football tournaments, youth clubs.	Need-supportive and need-thwarting behavior of parents	SDT	Coaching and parenting showed similar patterns of association, with the need to support being positively associated with autonomous motivation and engagement, while the need to discourage was positively associated with amotivation and disengagement. When considered together, the need for supportive coaching rather than nurturing was positively associated with autonomous motivation and engagement in football players.
De La Cruz et al. (2021)	Designed to examine the relationship between fathers’ expectations of their children’s youth baseball practice, their children’s basic psychological needs (satisfaction and frustration), and their willingness to continue or withdraw from baseball practice	Quant/Cross-Sectional	533 fathers (M = 44.30, SD = 5.18); 533 male adolescents (M = 13.09, SD = 1.68, AR = 10–16)	Mexico	Participation in regional competitions, youth baseball league.	Father’s intrinsic and extrinsic desires	SDT	The father’s intrinsic desire is positively associated with the satisfaction of the child’s psychological needs, while the father’s extrinsic desire is positively associated with the frustration of the child’s psychological needs.
Alvarez et al. (2021)	examine the controlling style in two contexts of social influence: the team (i.e., coach and teammates) and the family (i.e., father and mother), as well as the mediational role of motivation (autonomous, controlled, and amotivation) and its relationship with boredom and burnout in young swimmers.	Quant/Cross-Sectional	267swimmers (140F/127M) (Mage = 14.26, SD = 1.61, AR = 12–18)	Spain	Swimming club athletes at community, regional and national level	Control style of father, mother	SDT	Fathers’ control style was directly related to controlled motivation and burnout and indirectly related to boredom through the mediating role of swimmers’-controlled motivation. The association of mother’s control style with all variables studied was offset by father’s control interpersonal style.
O’Neil and Amorose (2021)	(a) identify different parenting styles of autonomous support and control based on perceptions of adolescent athletes, (b) examine the unique effects of parenting styles on adolescent athletes’ motivational responses (i.e., parent-to-child influence), and (c) understand the contribution of adolescent athletes’ motivation to the emergence of these parenting styles (i.e., child-to-parent influence).	Quant/longitudinal two-wave measurement	268 Athletes (96 M/172F AR = 14–18, Mage = 15.72, SD = 1.20)	United States	School sports team athletes	Parental autonomy support and control	SDT	The results provide evidence to support the four profile solution. The four parenting styles were found to differentially predict and anticipate adaptive and maladaptive motivational responses (i.e., basic psychological need satisfaction, autonomous and controlled motivation) in adolescent athletes. Parenting styles characterized by an autonomy-supportive dominant model were most appropriate for athletes’ motivation.
Krommidas et al. (2022)	The effects of coach-initiated motivational climate and parental support on intrinsic motivation, enjoyment of sport young football players’ participation, subjective dynamics, sport-related violence and academic achievement were examined. The second aim was to examine whether intrinsic motivation moderated the effects of coach-initiated climate and parental support on the above endogenous variables.	Quant/Cross-Sectional and longitudinal	T1: 494 footballers,471 M (23 N/a) AR = 8–15, Mage=: 11.51, SD = 1.58; T2: 188 footballers,182 M (6 N/a) AR = 9–15, Mage = 11.69, SD = 1.58	Greece	Youth football club player, competing in regional competitions.	Parental praise and understanding	SDT	Both a coach-initiated climate of empowerment and parental support lead to unique differences in intrinsic motivation and motivational outcomes. Parental praise and understanding, as well as coach support for athletes’ task engagement, autonomy and relevance, had a cumulative effect on athletes’ psychosocial development. Independent influences of coaches and parents may also occur. Coaches appear to have a greater influence than parents on the intrinsic motivation and sport-related peer environment of young football players.
McCann et al. (2021)	To identify the motivation-related influences perceived by coaches, parents and peers at all stages of the development of football players in the investment phase, and to determine how these influences change through the developmental stages.	Qual/Cross-Sectional	4 parents (3 M/1F); 4 M investment stage footballers (Mage = 18.5, SD = 0.6)	United Kingdom	Member of the English Elite Football Program	Parental interpersonal interaction, support for development, support for participation and feedback/assessment.	SDT/AGT/Motivational atmosphere model/	Coaches, parents and peers influence the motivation of football players in a number of ways, including the quality of their relationship with the player, their positive and negative behaviors, the support they provide for the player’s development and participation in football, and the support they provide for the footballer to reflect on their experiences. As athletes reach higher levels of performance, coaches and peers become more important.

Quant, Quantitative; Qual, qualitative; M, Male; F, Female; Mage, Mean Age; SD, Standard Deviation; AR, Age Range; N/a, Not available; CMT, competence motivation theory; AGT, achievement goal theory; SDT, self-determination theory; EVT, expectancy value theory.

Quality assessment of each article was conducted individually using the criteria proposed by Kmet et al. (2004). An assessment checklist was used for both qualitative and quantitative articles. For quantitative studies, a 14-item checklist was used to score each article based on the extent to which it met each criterion (2 = fully meets the standard, 1 = partially meets the standard, 0 = does not meet the standard). Items not applicable to specific research objectives were marked “n/a.” Quality assessment for qualitative studies was based on a 10-item checklist, using the same scoring scheme as for quantitative articles. The total quality score for each article was calculated using the relevant criteria and then converted to a percentage for standardization purposes. Scores of ≤55%, 55–75%, and ≥ 75% were considered low, medium, and high quality, respectively. Any low-quality studies were excluded from the systematic review (Kmet et al., 2004). The fifth and sixth authors assessed a random sample of both quantitative and qualitative studies along with their respective quality scores and deemed the results to be appropriate. The outcomes of our quality assessment procedures are presented in Tables 2, 3.

**Table 2:** Quality assessment of included quantitative studies.

Article	Quality assessment criteria														Total score	Quality score
	1	2	3	4	5	6	7	8	9	10	11	12	13	14		
Babkes and Weiss (1999)	Y	Y	P	P	n/a	n/a	n/a	Y	Y	Y	Y	n/a	Y	Y	18	90%
Given (2001)	Y	P	P	Y	n/a	n/a	n/a	Y	P	P	P	n/a	Y	Y	15	75%
Gagne (2003)	Y	Y	Y	Y	n/a	n/a	n/a	P	Y	Y	P	n/a	Y	Y	18	90%
White et al. (2004)	Y	Y	Y	Y	n/a	n/a	n/a	Y	Y	Y	Y	n/a	P	Y	19	95%
Waldron and Krane (2005)	Y	Y	Y	Y	n/a	n/a	n/a	P	P	Y	Y	n/a	P	Y	17	85%
Ullrich-French and Smith (2006)	Y	Y	Y	Y	n/a	n/a	n/a	Y	Y	Y	Y	n/a	Y	Y	20	100%
Papaioannou et al. (2008)	Y	Y	Y	Y	n/a	n/a	n/a	Y	Y	Y	Y	n/a	Y	Y	20	100%
Boiché et al. (2011)	Y	P	P	Y	n/a	n/a	n/a	P	Y	Y	P	n/a	Y	Y	16	80%
Sánchez-Miguel et al. (2013)	Y	Y	P	Y	n/a	n/a	n/a	P	Y	Y	P	n/a	Y	Y	17	85%
Hein and Jõesaar (2015)	Y	Y	Y	Y	n/a	n/a	n/a	Y	Y	Y	Y	n/a	Y	Y	20	100%
O’Rourke et al. (2014)	Y	P	P	P	n/a	n/a	n/a	Y	Y	Y	N	n/a	Y	Y	15	75%
Amado et al. (2015)	Y	Y	Y	Y	n/a	n/a	n/a	Y	Y	Y	Y	n/a	Y	Y	20	100%
Amorose et al. (2016)	Y	Y	Y	Y	n/a	n/a	n/a	Y	Y	Y	Y	n/a	Y	Y	20	100%
Schwebel et al. (2016)	Y	Y	Y	Y	n/a	n/a	n/a	Y	Y	P	Y	n/a	Y	Y	19	95%
Gaudreau et al. (2016)	Y	Y	P	Y	n/a	n/a	n/a	P	P	Y	Y	n/a	Y	Y	17	85%
Weltevreden et al. (2018)	Y	Y	Y	Y	n/a	n/a	n/a	Y	P	Y	Y	n/a	Y	Y	19	95%
Lienhart et al. (2019)	Y	Y	Y	Y	n/a	n/a	n/a	Y	Y	Y	Y	n/a	Y	Y	20	100%
Teques et al. (2019)	Y	Y	Y	Y	n/a	n/a	n/a	Y	Y	Y	Y	n/a	Y	Y	20	100%
Gomes et al. (2019)	Y	Y	P	Y	n/a	n/a	n/a	Y	Y	Y	Y	n/a	Y	Y	19	95%
Lienhart et al. (2020)	Y	Y	Y	Y	n/a	n/a	n/a	Y	Y	Y	Y	n/a	P	P	18	90%
Souza et al. (2020)	Y	Y	Y	P	n/a	n/a	n/a	Y	P	Y	P	n/a	P	Y	16	80%
De Muynck et al. (2021)	Y	Y	P	Y	n/a	n/a	n/a	P	Y	Y	Y	n/a	Y	Y	18	90%
De La Cruz et al. (2021)	Y	Y	Y	P	n/a	n/a	n/a	Y	Y	Y	Y	n/a	Y	Y	19	95%
Alvarez et al. (2021)	Y	Y	Y	Y	n/a	n/a	n/a	Y	Y	Y	Y	n/a	Y	Y	20	100%
O’Neil and Amorose (2021)	Y	Y	Y	Y	n/a	n/a	n/a	Y	Y	Y	P	n/a	Y	Y	19	95%
Krommidas et al. (2022)	Y	Y	P	Y	n/a	n/a	n/a	Y	Y	Y	P	n/a	P	Y	17	85%

(1) Question/objective sufficiently described? (2) Study design evident and appropriate? (3) Method of subject/comparison group selection or source of information/input variables described as appropriate? (4) Subject (and comparison group, if applicable) characteristics sufficiently described? (5) If interventional and random allocation was possible, was it described? (6) If interventional and blinding of investigators was possible, was it reported? (7) If interventional and blinding of subjects was possible, was it reported? (8) Outcome and (if applicable) exposure measure(s) well defined and robust to measurement/misclassification bias? means of assessment reported? (9) Sample size appropriate? (10) Analytical methods described/justified and appropriate? (11) Some estimate of variance is reported for the main results? (12) Controlled for confounding? (13) Results reported in sufficient detail? (14) Conclusions support the by results? Y, yes; P, partial; N, no; n/a, not applicable.

**Table 3:** Quality assessment of included qualitative studies.

Article	Quality assessment criteria										Total score	Quality score
	1	2	3	4	5	6	7	8	9	10		
Keegan et al. (2010)	Y	Y	Y	Y	Y	Y	Y	Y	Y	Y	20	100%
Felber Charbonneau and Camiré (2019)	Y	Y	Y	Y	Y	Y	Y	N	Y	N	16	80%
McCann et al. (2021)	Y	Y	Y	Y	Y	Y	Y	Y	Y	N	18	90%

(1) Question/objective sufficiently described? (2) Study design evident and appropriate? (3) Context for the study clear? (4) Connection to a theoretical framework/wider body of knowledge? (5) Sampling strategy described, relevant, and justified? (6) Data collection method clearly described and systematic? (7) Data analysis clearly described and systematic? (8) Use of verification procedure(s) to establish credibility? (9) Conclusions supported the by results? (10) Reflexivity of the account? Y, yes; P, partial; N, no.

## Results

### Study characteristics

Table 1 summarizes the characteristics of the 29 papers that met the criteria for inclusion in this review. The final sample consisted of 26 quantitative papers and 3 qualitative papers. The studies employed cross-sectional (82.8%) and longitudinal (17.2%) research designs, collectively involving 9,185 young athlete participants and 2,191 parent participants. In assessing the gender of these athlete and parent participants, it was observed that there were 6,055 (65.9%) male and 3,074 (33.5%) female young athlete participants, with 56 (0.6%) not reporting gender; among parent participants, 1,175 (53.6%) were male, 695 (31.7%) female, and 321 (14.7%) did not report gender. Qualitative studies involved a total of 91 young athlete participants and 12 parent participants, whereas quantitative studies involved 9,094 young athlete participants and 2,179 parent participants. Out of all the studies, 8 (28%) included data collection through parents, with quantitative (*n* = 6) and qualitative (*n* = 2) approaches. The remaining 21 (72%) studies collected data solely through athletes. The ages of the young athletes ranged from 8 to 22, with 15 (51.7%) studies focusing solely on early to mid-adolescents (up to 16 years old). Fourteen (48.2%) studies covered late adolescence or the entire adolescent period. The young athletes were recruited from various levels of sports clubs (*n* = 17), school sports teams (*n* = 5), specific competitive sports programs (*n* = 4), national training centers (*n* = 2), and one study did not report the source (*n* = 1).

Among the 29 studies, some investigated single sports, including basketball (Schwebel et al., 2016; Souza et al., 2020), soccer (Babkes and Weiss, 1999; Ullrich-French and Smith, 2006; De Muynck et al., 2021; McCann et al., 2021; Krommidas et al., 2022), baseball (De La Cruz et al., 2021), swimming (Given, 2001; O’Rourke et al., 2014; Alvarez et al., 2021), softball (Waldron and Krane, 2005), and gymnastics (Gagne, 2003), among others. The other 16 studies explored a variety of sports ranging from individual sports (e.g., athletics, tennis, swimming, gymnastics, tennis, judo) to team sports (e.g., basketball, football, volleyball, baseball, softball, soccer). The competition levels of the athletes varied within and between studies, including recreational, regional, state, national, and international levels. Most studies were conducted in the United States (*n* = 9), followed by the United Kingdom/France/Spain (*n* = 3), Greece/Canada/Portugal (*n* = 2), and Brazil/Belgium/Mexico/Estonia/Netherlands (*n* = 1).

### Parental goals and values

A total of six quantitative studies discussed the relationship between Parental Goals and Values and the motivation of young athletes. These studies highlight that under the Achievement Goal Theory, both Task orientation and Ego orientation of young athletes are highly correlated with their parents’ goal orientations, regardless of whether the evidence comes from athletes’ reports (Given, 2001) or parents’ subjective reports (Sánchez-Miguel et al., 2013). Additionally, the belief that parents’ efforts lead to sports success (White et al., 2004) and perceived parent mastery success standards were significantly related to young athletes’ Task orientation (Schwebel et al., 2016). The belief that parental ability, external factors, and deception lead to sports success (White et al., 2004) and Perceived parental ego success standards (Schwebel et al., 2016) were significantly related to Ego orientation. Moreover, parental success standards moderated the relationship between the motivational climate initiated by coaches and achievement motivation (Schwebel et al., 2016).

Under the framework of Self-Determination Theory, fathers’ self-reported intrinsic aspirations are related to the satisfaction of basic psychological needs, while their self-reported extrinsic aspirations are related to the frustration of these needs (De La Cruz et al., 2021).

According to Competence Motivation Theory, more positive parental beliefs about a child’s abilities are positively correlated with higher perceived competence and intrinsic motivation in athletes (Babkes and Weiss, 1999).

Overall, parents’ positive beliefs and values are associated with positive motivational variables in athletes, and parents’ goals and values influence young athletes’ motivation in conjunction with coaches.

### Parenting styles

Eight quantitative studies and one qualitative study have discussed the relationship between parental parenting styles and the motivation of young athletes. In the realm of Achievement Goal Theory, perceived parental psychological control positively influences adolescents’ task orientation, while perceived parental responsiveness yields opposite results (Weltevreden et al., 2018). Moreover, perceived parental behavioral control correlates positively with ego orientation, while perceived parental autonomy support has an inverse effect (Weltevreden et al., 2018).

In the context of Self-Determination Theory, parental autonomy support is a focal point of current research. Both the perceived autonomy support from parents (Hein and Jõesaar, 2015) and the individual autonomy support from each parent have shown significant correlations with the Self-Determined Motivation Index (Amorose et al., 2016). Additional evidence also underscores a notable relationship with the index of autonomous regulation and self-determined sport motivation (Gagne, 2003; Gaudreau et al., 2016; O’Neil and Amorose, 2021). Regarding basic psychological needs, perceived parental autonomy support positively impacts the fulfillment of these needs (Gaudreau et al., 2016), although earlier studies only provided evidence concerning relatedness (Gagne, 2003). Moreover, strong autonomy support from both parents represents the optimal parenting approach for fostering adaptive motivational outcomes in young athletes (O’Neil and Amorose, 2021). This relationship underscores the importance of parents’ supportive behavior in fostering self-determined motivation in young athletes.

In considering the influence of coaches, interactive effects have also been observed. Amorose et al. (2016) found that autonomy support from significant social agents independently predicts self-determined motivation and can also synergistically enhance this prediction. Gaudreau et al. (2016) discovered that parental autonomy support modulates the impact of coach autonomy support. Hein and Jõesaar (2015) also noted that autonomy support from parents is a stronger predictor of self-determined motivation than that from coaches. Overall, a parenting style centered on autonomy support correlates positively with motivational variables, but it’s crucial to consider the interactive effects between other significant social agents and both parents.

In contrast, the perceived controlling styles of both fathers and mothers are significantly related to controlled motivation (introjected and external regulation) and amotivation. However, the mother’s controlling style is offset by the father’s interpersonal control (Alvarez et al., 2021). O’Neil and Amorose (2021) reported that weak control from both parents represents the most detrimental parenting style. Parental control can coexist with autonomy support without diminishing its positive impact on self-determined motivation (O’Neil and Amorose, 2021).

Qualitative research has yielded similar findings, indicating that autonomy support is generally considered to have a positive impact on motivation. Conversely, a controlling style is often associated with feelings of depression, anger, diminished motivation, and even the breakdown of relationships (Keegan et al., 2010).

### Parental involvement behaviors

A total of 12 quantitative studies and 3 qualitative papers have discussed the relationship between parental involvement behaviors and motivational variables. In the realm of Achievement Goal Theory, parents’ self-reported supportive and understanding behaviors (Sánchez-Miguel et al., 2013), as well as perceived maternal sports support and both maternal and paternal sports expectations (Gomes et al., 2019), are significantly correlated with Task Orientation. Interestingly, perceived maternal competition attendance shows an inverse relationship with Task Orientation (Gomes et al., 2019). Additionally, parents’ self-reported directive behaviors and pressure (Sánchez-Miguel et al., 2013), along with perceived paternal and maternal performance pressure and sports expectations (Rui Gomes et al., 2019), are significantly related to Ego Orientation. Moreover, research indicates that athletes’ cognitions may modulate the impact of parental behaviors (Gomes et al., 2019).

In line with Self-Determination Theory, athletes’ perception of parental need-supportive behaviors is significantly associated with self-determined sport motivation (De Muynck et al., 2021). Conversely, perceived parental need-thwarting behaviors and parental pressure are strongly linked to controlled motivation (Lienhart et al., 2019). It is worth noting that moderate parental involvement has an inverse relationship with controlled motivation (Lienhart et al., 2020). Moreover, research indicates that positive parental behaviors, such as perceived involvement, praise, and understanding, robustly correlate with intrinsic motivation (Gagne, 2003; Teques et al., 2019; Krommidas et al., 2022). In contrast, negative parental behaviors, including self-reported pressure and perceived need-thwarting actions, are significantly related to amotivation (Sánchez-Miguel et al., 2013; Amado et al., 2015; De Muynck et al., 2021). Notably, a counterintuitive finding suggests that perceived maternal pressure can positively predict intrinsic motivation and identified regulation (Lienhart et al., 2019).

Similar findings are also evident in the context of Basic Psychological Needs. For instance, self-reported parental pressure negatively predicts athletes’ satisfaction of Basic Psychological Needs (Amado et al., 2015). Perceived parental praise and understanding (Lienhart et al., 2019), as well as moderate parental involvement (Lienhart et al., 2020), positively predict satisfaction in the domains of competence and relatedness. Conversely, perceived parental pressure yields negative predictions for these outcomes (Lienhart et al., 2019). Additionally, moderate parental involvement is inversely correlated with the thwarting of basic psychological needs for autonomy (Lienhart et al., 2020).

Some specific differences have been emphasized in the research. In terms of gender differences, Lienhart et al. (2019) found that the direction of the relationship between introjected regulation and perceptions of paternal guidance and maternal pressure varies between boys and girls. More significant relationships were observed between boys and their same-sex parents, with boys’ outcomes primarily related to parental behavior. Negative predictions from parents were also found to be stronger than positive ones (Lienhart et al., 2019). Similar results were reflected in male athletes experiencing greater parental pressure (Amado et al., 2015; O’Neil and Amorose, 2021). Regarding athlete-level differences, significant variations were found in the impact of perceived parental encouragement and role-modeling on intrinsic motivation between elite and sub-elite athletes (Teques et al., 2019). In considering the differential impact of coaches and parents, supportive coaching, rather than parenting, was positively correlated with soccer players’ autonomous motivation and engagement, while thwarting coaching and parenting were positively correlated with amotivation (De Muynck et al., 2021).

Research based on Expectancy-Value Theory suggests that maternal directive behavior negatively impacts athletes’ perceived competence, while maternal praise and understanding are positively correlated with intrinsic motivation. Additionally, perceived praise and understanding from mothers and positive involvement from fathers are positively associated with value (Boiché et al., 2011). Studies grounded in Achievement Motivation Theory indicate that parents perceived as athletic role models, who offer frequent positive contingent responses to successful performance, are positively correlated with athletes’ higher perceived competence and intrinsic motivation (Babkes and Weiss, 1999).

Qualitative research emphasizes that parental autonomy-supportive behavior can provide young athletes with a sense of autonomy, competence, and relatedness. In contrast, controlling behavior is considered to inhibit psychological needs. Mixed parental behavior is thought to both satisfy and frustrate some of the children’s basic psychological needs (Felber Charbonneau and Camiré, 2019). McCann et al. (2021) highlight that parental supportive behavior enhances athletes’ intrinsic motivation. Various forms of support, such as tangible, effort-based, social support, parental evaluation (supportive reflection and prospective planning), and feedback (praise), also maintain and protect athletes’ motivation. Keegan et al. (2010) stress that positive feedback (constructive feedback) is considered to generate more adaptive motivation, while negative feedback (summative feedback) is more likely to destroy motivation and induce frustration. Unconditional praise from parents positively impacts motivation, and parents’ pre-competition motivational behavior promotes effort/mastery, stress/avoidance, and confidence/approach motivations (Keegan et al., 2010).

In summary, parents’ positive involvement—comprising support, praise, understanding, and attendance at competitions—facilitates favorable motivational outcomes in young athletes. Conversely, negative involvement, characterized by directive behavior, pressure, and performance expectations, correlates with adverse motivational outcomes. Moderate parental involvement is considered the optimal level of engagement.

### Parent-initiated motivational climate

In summary, three quantitative studies have discussed the impact of the motivational climate initiated by parents on adolescent athletes’ motivation. Under the framework of Achievement Goal Theory, it is emphasized that a parental atmosphere focusing on learning and enjoyment (Waldron and Krane, 2005), as well as a task climate initiated by parents (Weltevreden et al., 2018), positively correlates with Task Orientation. An ego climate initiated by parents shows a positive correlation with Ego Orientation (Weltevreden et al., 2018). Notably, the motivational climate initiated by parents can mediate the relationship between general parenting behavior and achievement motivation (Weltevreden et al., 2018).

In the context of Self-Determination Theory, a perceived task climate from parents is positively correlated with the index of autonomous regulation, while a perceived ego climate offers an inverse relationship (O’Rourke et al., 2014).

Overall, the task climate initiated by parents significantly influences young athletes’ achievement motivation and self-determined motivation.

### Parent–child relationships

A quantitative study by Ullrich-French and Smith (2006) discussed the relationship between parent–child relationships and motivational outcomes. Under Self-Determination Theory, they discovered that perceived positive mother–child and father–child relationship quality is significantly related to the Self-determined Motivation Index. This suggests a strong correlation between positive parent–child relationships and autonomous motivation. Both mother–child and father-child relationships significantly predict self-determined motivation, and it has also been found that parents and peers may influence self-determined motivation in both additive and collective ways (Ullrich-French and Smith, 2006). This highlights the multifaceted nature of influences on young athletes’ motivation, where the roles of both familial and peer relationships are crucial. In the context of Competence Motivation Theory, perceived positive father–child relationship quality was significantly related to athletes’ perceived competence (Ullrich-French and Smith, 2006). However, the quality of father-child or mother–child relationships alone could not predict athletes’ perceived competence. Overall, positive parent–child relationships are key in fostering positive motivational outcomes in young athletes.

## Discussion

The objective of this systematic review is to summarize the empirical evidence on the role of parents in the motivation of young athletes and to provide practical insights and recommendations for future research. A total of 29 studies, both quantitative and qualitative, were reviewed. A comprehensive review of the literature reveals the unique and synergistic multi-dimensional roles that parents play in the motivation of young athletes. Optimal parenting strategies are identified as those that incorporate positive goals and values, an autonomy-supportive parenting style, moderate levels of parental involvement, positive parent–child relationships, and a parent-initiated task climate.

### Parental goals and values

Current evidence consistently shows a significant correlation between parents’ goal orientations and athletes’ goal orientations (Given, 2001; Papaioannou et al., 2008; Sánchez-Miguel et al., 2013). However, earlier studies indicated that young athletes’ goal orientations were only related to athletes’ self-reported perceptions of their parents’ goal orientations (Given, 2001). This may be attributed to factors such as smaller sample sizes or specific sports types (swimming) and possibly specific socioeconomic backgrounds of the samples. In contrast, broader sports types and larger sample sizes produced consistent results between parents’ reported goal orientations and athletes’ self-reported goal orientations (Sánchez-Miguel et al., 2013). Additionally, the possibility of adults not explicitly conveying their value systems related to goals to adolescents, or making false statements about their goals, could also contribute to discrepancies in smaller sample studies. Future research could provide more detailed explanations from aspects like socioeconomic status and types of sports (individual or collective). This would further our understanding of the impact of parents’ words and actions.

In summary, this aligns with previous findings in the field of parenting, where parents are seen as primary role models for their children (Wiese and Freund, 2011). Their goals and values are often internalized by children (i.e., imitating parents’ behaviors and values), thereby influencing their motivation (Anderson and Cavallaro, 2002). Additionally, parents’ expectations can shape children’s intrinsic motivation (Yamamoto and Holloway, 2010). That is, if parents have high expectations for a certain behavior in their child, this can further promote effective parental involvement and also foster the child’s intrinsic motivation and self-efficacy (Yamamoto and Holloway, 2010). Lastly, parents’ values have a significant impact on the socialization process of children (Barni et al., 2017). The values held by parents often influence their parenting styles, which in turn affect the motivational behavior of children (Bouissou and Tap, 1998).

The findings of this review emphasize that parents’ goals and values do not always have a positive impact on their children (Babkes and Weiss, 1999; Schwebel et al., 2016; De La Cruz et al., 2021). This could be because excessive expectations or pressure may undermine children’s sense of self-efficacy, leading to decreased motivation (Yamamoto and Holloway, 2010). Similarly, if parents’ values conflict with children’s self-perceptions, it may affect their motivation (Knafo and Assor, 2007; Moed et al., 2015). Therefore, understanding and balancing parents’ goals and values are crucial for fostering positive motivation in children.

Furthermore, parents’ success standards have been found to moderate the relationship between the motivational climate initiated by coaches and motivation (Schwebel et al., 2016). This indicates that the most important social agents for athletes can collaboratively create an environment through their motivational climates, thereby influencing the quality of their sports experiences and well-being (Henriksen et al., 2020). However, further exploration is needed regarding the potential interactive influences of other significant social agents, such as siblings, peers, and teachers (Garcia Bengoechea and Strean, 2007).

### Parenting styles

The results of this review emphasize that parental autonomy support promotes positive motivational outcomes and is particularly relevant in the context of self-determination (Gagne, 2003; Keegan et al., 2010; Hein and Jõesaar, 2015; Amorose et al., 2016; Gaudreau et al., 2016; O’Neil and Amorose, 2021). Research from a number of parenting fields supports our results that pro-autonomous parenting is characterized by the provision of a supportive environment in which parents understand, acknowledge and support their adolescents’ feelings and perspectives (McCurdy et al., 2020). Parents who adopt this approach encourage autonomous action and decision-making, fostering intrinsic motivation in their children (Soenens et al., 2007; Zhou et al., 2019). Parental autonomy support enhances self-confidence, enjoyment of exercise, and determination and perseverance to overcome challenges (Furusa et al., 2021; Gao et al., 2021; Du et al., 2023). Additionally, parental autonomy support mitigates reactions to maladaptive outcomes and promotes emotional regulation skills, thereby promoting children’s psychological well-being, resilience, and long-term engagement (Cheung and Pomerantz, 2011; Simon, 2021; Zeng et al., 2022).

In contrast, a controlling parenting style has been shown to yield negative motivational outcomes (Keegan et al., 2010; Alvarez et al., 2021). Such parents dictate behaviors and outcomes, exerting pressure and criticism, which disrupts adolescents’ needs for autonomy, relatedness, and competence (Soenens and Vansteenkiste, 2010). This high level of intrusion into the personal domain of adolescents inevitably leads to adverse psychological outcomes (Nucci et al., 2005). This approach often violates adolescents’ self-perception, making them feel as if they are fulfilling others’ expectations rather than pursuing their own interests (Barber and Harmon, 2002). Moreover, a controlling parenting style may cultivate a maladaptive focus on outcomes rather than the learning process (Aunola et al., 2000; Hibbard and Walton, 2014), which could hinder skill development and enjoyment in sports (Mallinson-Howard et al., 2019; Morano et al., 2022).

Parental autonomous support can interact with coaching styles in ways such as synergy, compensation, and moderation (Hein and Jõesaar, 2015; Amorose et al., 2016; Gaudreau et al., 2016). This aligns with the principles of positive youth development, which emphasize that when young athletes in sports environments receive appropriate support from others, it ensures more positive developmental outcomes and sustained participation in youth sports (Holt et al., 2017). However, the interactive effects between parental autonomous support and other significant social agents remain an area with research gaps. Additionally, the simultaneous occurrence of parental control alongside parental autonomous support (O’Neil and Amorose, 2021) and its role in the overall motivational climate for young athletes warrant further exploration. This line of inquiry could provide deeper insights into the complex dynamics of parental influence in the context of youth sports.

### Parental involvement behaviors

Parental involvement in the sports environment is one of the most direct and profound ways to influence the psychological and social development of young athletes (Knight, 2019). The results of this review emphasize that positive parental involvement behaviors (such as support, praise, understanding actions, and competition participation) can promote positive motivational outcomes in young athletes. In contrast, negative involvement (such as directive behavior, pressure, and expectations related to sports) can lead to adverse outcomes (Babkes and Weiss, 1999; Gagne, 2003; Keegan et al., 2010; Boiché et al., 2011; Sánchez-Miguel et al., 2013; Felber Charbonneau and Camiré, 2019; Gomes et al., 2019; Lienhart et al., 2019; Teques et al., 2019; Lienhart et al., 2020; De Muynck et al., 2021; McCann et al., 2021; O’Neil and Amorose, 2021; Krommidas et al., 2022). Positive parental involvement helps young athletes build stronger self-confidence, enhances their ability to cope with competitive stress, and fosters a love for and commitment to the sport (; Furusa et al., 2021; Rouquette et al., 2021). In our research findings, Lienhart et al. (2020) discovered that a moderate level of parental involvement is most beneficial for the development of athletes throughout the sports season, particularly in sub-elite and elite athletes. However, this result may have limited applicability in different sports contexts or demographic groups due to the specific level of sports (sub-elite and above) and cultural background (France) of the study. Nevertheless, as other studies have shown, excessive positive involvement and pressure from parents can cause stress and discomfort in children, and adolescents tend to prefer parental involvement characterized by praise and understanding (Bonavolontà et al., 2021; Coutinho et al., 2021). Future research could further explore this in different cultural contexts and among young athletes at various stages of their sports careers.

Additionally, our review yielded some counterintuitive findings regarding perceived maternal involvement in competitions (Gomes et al., 2019) and perceived pressure from mothers (Lienhart et al., 2019). These anomalies could be attributed to mothers’ lack of sports knowledge or to the unpredictable factors during competitions (Clarke et al., 2016). Gender differences in the outcomes of parental involvement (Amado et al., 2015; Lienhart et al., 2019; O’Neil and Amorose, 2021) may be rooted in the traditional roles fathers and mothers play in various cultures (Wall and Arnold, 2007; Waters et al., 2022). In youth sports, mothers are more inclined to offer nurturing and emotional support, while fathers are more likely to engage in physical activities and provide opportunities for exploration and adventure (Lindstrom Bremer, 2012). Fathers may have higher expectations for their children’s athletic success, particularly for boys, whereas mothers may prioritize their children’s physical health and safety (Coakley, 2006; Gottzén and Kremer-Sadlik, 2012; Lindstrom Bremer, 2012). Further research could examine the dynamics of the relationship between athletes of different genders and their parents in a wider sporting context.

Moreover, our results indicate that negative parental behaviors have a stronger predictive power than positive ones (Lienhart et al., 2019; De Muynck et al., 2021). In a sports context, negative parental actions can indeed lead to highly unsettling and hard-to-ignore phenomena, such as conditional respect and off-field anger (Goldstein and Iso-Ahola, 2008; Ross et al., 2015). Future research on interventions to optimize parental education in sports may need to pay additional attention to this aspect (Knight et al., 2017).

### Parent-initiated motivational climate

The results of this review emphasize that parent-initiated task climate is associated with positive motivational outcomes (Nucci et al., 2005; O’Rourke et al., 2014; Weltevreden et al., 2018). The term ‘motivational atmosphere’ was coined by Keegan et al. (2011) to reflect the intricate complexity of the social environment in shaping athlete motivation. Parents create a motivational climate that is shaped by parental behaviors, words, expectations and feedback, which together determine the level of support and pressure a child feels (O’Rourke et al., 2012; Harwood et al., 2015; Frydrychová et al., 2017). Task climate emphasizes the importance of characteristics (e.g., effort, enjoyment, proficiency) that are more susceptible to the athlete’s personal control than an ego climate that emphasizes winning, losing, or being superior to others (Granero-Gallegos et al., 2017). In summary, when children take pride in non-normative progress, internal self-reinforcement processes may be engaged and amplified, thereby fostering positive motivational outcomes (Rourke and Smith, 2013). However, current research has not explored more complex multidimensional models of achievement goals, such as the 3 × 2 model (Elliot et al., 2011), which warrants further investigation.

### Parent–child relationships

Based on our systematic review, a positive parent–child relationship significantly enhances the motivational outcomes in adolescent athletes (Ullrich-French and Smith, 2006). Interpersonal relationships are a crucial factor in influencing an athlete’s motivation (Garcia Bengoechea and Strean, 2007). The closeness and security in parent–child relationships provide a stable foundation for young athletes, making them feel supported and understood (Lisinskiene et al., 2018). Furthermore, a positive parent–child relationship is associated with better communication (Lippold et al., 2016), which not only increases positive sports feedback from parents but also encourages a more autonomy-supportive parenting style (Azimi and Tamminen, 2022). Additionally, strong parent–child relationships cultivate a sense of competence and autonomy in athletes, thereby increasing intrinsic motivation and enjoyment in sports (Rouquette et al., 2020). The review also found that parents and peers could influence self-determined motivation in a cumulative and collective manner (Ullrich-French and Smith, 2006). This is because the social influences in sports can come from multiple sources, including parents, peers, siblings, coaches, and fans, affecting choices, attitudes, and behaviors in sports (Partridge, 2011). Furthermore, in the interpersonal environment of youth sports, the relationship between coaches and parents is also considered an important factor and warrants further comprehensive consideration (Harwood et al., 2019).

## Limitations

The limitations of this systematic review include its focus on English-language articles, potentially overlooking studies in other languages. The majority of the reviewed literature comes from Western cultures, limiting the applicability of findings to diverse cultural contexts. Since each parent can have a different impact on motivation depending on their relationship with the child, sports experience, or emotional style, and this relationship may provide contextual background for the child’s motivation (Holt et al., 2008). Many studies used cross-sectional designs, which cannot establish causality, and relied on surveys and interviews, possibly leading to response bias or recall errors. Despite a comprehensive literature search, it’s still possible that some studies relevant to the topic were missed due to selection criteria or other factors. Furthermore, as the included studies did not distinguish between stages of sports participation and age groups, the review does not differentiate how the relationship between parents and young athletes’ motivation may vary dynamically at different stages. The stages of sports participation also differ between different sports, posing a challenge to distinguish parental influence on young athletes by age.

## Proposals for future research

Future research should broaden the sample scope to include populations from diverse cultural and socio-economic backgrounds and encompass a variety of sports, including adventure, extreme, and winter sports among youth athletes. It is recommended to use longitudinal, experimental, and mixed-methods research designs to delve into the dynamics of parental influence over time. Comprehensive studies on parental influence methods should also be conducted, considering the implementation of parental education and intervention programs, such as utilizing modern technology (mobile apps, online platforms). Moreover, future studies should evaluate the effectiveness of different parenting attitudes, styles, and behaviors, which may vary according to the child’s developmental stage, gender, type of sport, and level of competition. Particularly at different developmental stages of children (e.g., from childhood to adolescence), parental influence may change with the increasing impact of coaches, teammates, and peers. Considering the functionality of sports types (individual vs. team) and levels of competition (recreational, competitive, elite), parental influence may also differ. These research directions will contribute to a more comprehensive understanding of the dynamic relationship between parents and young athletes’ motivation and provide guidance for the development of effective parental involvement and support strategies in sports.

## Conclusion

This systematic review synthesizes research evidence from four theoretical backgrounds to explore the pivotal role parents play in shaping the motivation of young athletes. Our findings underscore the impact of parental goals and values, parenting styles, involvement behaviors, created motivational climates, and parent–child relationships on the motivational outcomes of young athletes. In summary, while parents undeniably play a crucial role in motivating young athletes, the manner and extent of their involvement are key.

## Data availability statement

The original contributions presented in the study are included in the article/supplementary material, further inquiries can be directed to the corresponding author.

## Author contributions

ZG: Conceptualization, Data curation, Investigation, Methodology, Project administration, Software, Visualization, Writing – original draft, Writing – review & editing, Formal analysis, Supervision. CC: Conceptualization, Data curation, Investigation, Methodology, Project administration, Supervision, Validation, Writing – review & editing, Writing – original draft. MN: Investigation, Supervision, Validation, Writing – review & editing, Data curation, Writing – original draft. JW: Data curation, Investigation, Methodology, Validation, Writing – review & editing. XZ: Data curation, Formal analysis, Investigation, Methodology, Writing – review & editing. TW: Data curation, Investigation, Methodology, Writing – review & editing.
